# Real-time acoustic sensing and artificial intelligence for error prevention in orthopedic surgery

**DOI:** 10.1038/s41598-021-83506-4

**Published:** 2021-02-17

**Authors:** Matthias Seibold, Steven Maurer, Armando Hoch, Patrick Zingg, Mazda Farshad, Nassir Navab, Philipp Fürnstahl

**Affiliations:** 1 Computer Aided Medical Procedures (CAMP), Technical University of Munich, 85748 Munich, Germany; 2 Research in Orthopedic Computer Science (ROCS), University Hospital Balgrist, University of Zurich, Balgrist Campus, 8008 Zurich, Switzerland; 3 Balgrist University Hospital, 8008 Zurich, Switzerland

**Keywords:** Preclinical research, Computer science, Musculoskeletal system, Biomedical engineering

## Abstract

In this work, we developed and validated a computer method capable of robustly detecting drill breakthrough events and show the potential of deep learning-based acoustic sensing for surgical error prevention. Bone drilling is an essential part of orthopedic surgery and has a high risk of injuring vital structures when over-drilling into adjacent soft tissue. We acquired a dataset consisting of structure-borne audio recordings of drill breakthrough sequences with custom piezo contact microphones in an experimental setup using six human cadaveric hip specimens. In the following step, we developed a deep learning-based method for the automated detection of drill breakthrough events in a fast and accurate fashion. We evaluated the proposed network regarding breakthrough detection sensitivity and latency. The best performing variant yields a sensitivity of $93.64 \pm 2.42$% for drill breakthrough detection in a total execution time of 139.29${\hbox { ms}}$. The validation and performance evaluation of our solution demonstrates promising results for surgical error prevention by automated acoustic-based drill breakthrough detection in a realistic experiment while being multiple times faster than a surgeon’s reaction time. Furthermore, our proposed method represents an important step for the translation of acoustic-based breakthrough detection towards surgical use.

## Introduction

Surgical interventions are conducted by trained and experienced experts, however, human errors are inevitable. In the operating room, surgical errors can lead to significant and severe consequences for the patient, in the worst case to death^1^. Prior studies showed that surgical factors account for more than 70% of intraoperative complications^2,3^. For example in orthopedic surgery, iatrogenic femoral arterial^4^ and nerve^5^ injury are frequently happening complications caused by surgical errors. Detecting and preventing these incidents is crucial to improve the patient safety and the outcome of surgery^6^.

There is a variety of causes for surgical errors and resulting iatrogenic injuries. They range from anatomical differences between patients and proximity of risk structures^7^ or lack of surgical access and overview, for example in obese patients^8^, to pathologically altered tissue substance, e.g. in patients with osteoporosis^9^. Furthermore, the condition of the surgeon and the surgical staff plays an important role on the performance and therefore the outcome of the surgery, as lack of concentration and technical incapacity can lead to an increased risk of iatrogenic injury^10^.

To assess the patient-specific risk of treatment complications in orthopedic surgery, commonly a pre-operative plan based on the patient anatomy and medical imaging data, such as radiographs, computed tomography (CT) or magnetic resonance imaging (MRI)^11^, is made. Furthermore, specialized imaging modalities, for example angiography^7^ or ultrasound^12,13^ are utilized to visualize anatomical risk structures such as nerves and arteries. Conventional navigation systems provide a way to transition pre-operative information into surgery by displaying it in relation to intraoperative information on an external monitor^14^ or even actively guide the surgeon through robotic assistance^15^, but prompt a need for additional optical tracking systems and time-consuming registration procedures which can introduce additional errors by registration failures^16^. Learning-based systems have great potential to support the surgeon during the intervention and enable augmented decision making based on real-time sensor data and additional learned knowledge^17–19^. They can be employed for active error prevention by detecting surgical states and important or adverse events during surgery in an automated fashion.

A relevant target task for an error prevention system in surgery is drill breakthrough detection. Drill breakthrough is defined by the drill perforating the bone and over-drilling beyond the far cortex into the adjacent soft tissue. With the rise of machine learning methods, learning-based techniques also have been applied for the task of automated drill state and breakthrough detection using real-time sensor data and achieved promising results. Bone drilling is an essential part of orthopedic surgery and is conducted in about 95% of the interventions, for example to fixate bones with plates, external fixators and traction equipment^20^. One of the most common ways of drilling in orthopedic procedures is to use free-hand power drills to manually pre-drill holes for bone screws. A study^21^ investigated free-hand drilling with a total number of 153 participating surgeons and found the average penetration of the soft tissue beyond the far cortex to be 6.31 mm which implies a great risk, especially when nerves, vessels or other vital structures are situated in close proximity to the target anatomy. The most important factor to stop the hand-operated drill as soon as possible after a breakthrough is the human reaction time. Even though trained surgeons have comparatively fast reaction times, their mean response time was measured to be in the range of 313 to 358${\hbox { ms}}$ which additionally decreases with advancing age^22^. A low-latency and robust detection system could enable a fast and automated stopping of the drill as soon as a breakthrough event is detected.

Drilling into a bone creates distinct vibrations resulting in the generation of acoustic signals which can be exploited for drill state and breakthrough analysis and have benefits over force/torque or current measurement approaches, such as easy integration and general applicability. Praamsma *et al.* showed in a study that experienced surgeons benefit from these audible sounds by utilizing them to support the drilling process^23^. In this work, we used a custom piezo contact microphone to capture drill vibration signals in an experimental setup and propose a fast and robust deep-learning based drill breakthrough event detection method. The key contributions of our work are:

- We developed a custom high-sensitive piezo-based contact microphone prototype and impedance matching / pre-amplification stage for capturing structure-borne drill vibration signals non-invasively from the skin surface.
- We propose a low-latency and robust deep learning-based drill breakthrough detection method based on a modified ResNet-18 architecture, handling imbalanced data through the application of the *Focal Loss* function.
- We trained and validated our method on a dataset captured in an experimental setup using 6 unprepared human cadaveric hips including soft tissue.
- The proposed method outperforms the results of prior studies (using artificial bone models or prepared animal bone specimens) in a realistic cadaveric experiment.

### State-of-the-art in acoustic-based drill breakthrough and drill state detection

Acoustic signals have been analyzed in prior work in order to detect both, drill breakthrough (penetration from bone into soft tissue) and drill state (type of tissue being drilled), using synthetic and animal bone models. The following paragraph gives an overview of the state-of-the-art in acoustic-based drill breakthrough and drill state detection and the transition from signal processing approaches to learning-based solutions in recent years.

One possible approach to implement automated drill state and breakthrough detection is based on force and torque measurements. Force/torque sensors offer reliable and accurate measurements of the force between drill bit and tissue and are therefore well suited for drill state and breakthrough detection^24–26^. Recently, Torun *et al.* proposed a closed-loop method based on force sensor data to detect breakthrough events in an experimental setup operating on a sheep femur^27^. As explained in their follow-up work^28^, this approach has disadvantages for the application in real surgery, because they require physical modifications to the surgical device, in form of sensors attached to the drill which are costly and bulky. Another approach has been proposed to detect breakthrough events in electric drills by measuring changes in the current flow through the motor^29^, which is however not suited for all types of surgical drills, such as pneumatic drills.

Because bone tissue consists of substructures with different density (cortical bone, cancellous bone and bone marrow), the friction between drill bit and tissue results in force and torque differences^30^ and therefore in distinct vibrations for different tissue types during bone drilling. Drill breakthrough events result in an abrupt vibration change when perforating from high density cortical bone into soft tissue surrounding the bone. These distinct vibrations can be measured as acoustic signals. Therefore, acoustic-based drill state and breakthrough detection have been proposed in the literature as a low-cost and easy-to-integrate alternative to force/torque-based solutions.

Acoustic-based drill state detection has been introduced to classify different types of bone tissue during drilling by analyzing audio signals recorded from the area of operation. The first approaches achieved this task using signal processing-based techniques. A power spectral density based classification system was introduced by Sun *et al.* and evaluated in an experimental setup with five porcine scapulae using an air-borne room microphone^31^. Yu *et al.* proposed a sound-based solution for distinguishing between cortical and cancellous bone during surgical milling utilizing a wavelet package transform energy based state identification^32^.

Furthermore, learning-based approaches have been proposed to classify drill vibration signals based on prior knowledge. Boesnach *et al.* developed a method to analyze drill sounds in spine surgery by applying neural networks, support vector machines (SVM) and Hidden Markov Models (HMM)^33^ to spectral density estimates. Zakeri *et al.* developed an experimental setup and learning-based classification method to distinguish between cortical and cancellous bone using six bovine tibiae by analyzing air-borne acoustic signals captured with a microphone^34^. Their method is based on short-time Fourier transform (STFT) features in combination with a SVM classifier which achieved accuracies of up to 83%. In their follow-up research, they investigated different logistic regression, SVM, random forest (RF) and HMM classifiers and compared time and frequency features in regard to classification performance. The highest average accuracy of 84.3% could be achieved by using wavelet packet transform features^35^. For the application in pedicle screw placement, a state recognition approach with handcrafted features and a neural network classifier was proposed by Guan *et al.*^36^. They showed that the detection of different bone layers using acoustic emission signals is more accurate and precise compared to force/torque measurements in a bovine test specimen. The recognition rate was reported as 84.2%.

The task of drill breakthrough detection differs from the drill state classification problem, as short breakthrough events have to be detected with high accuracy and as fast as possible. The aim is here to stop the drill after perforating the cortical bone to avoid damage to surrounding soft tissue. An automatic method to stop the drill when perforating a rat skull based on spectral density features and a SVM classifier was proposed by Pohl *et al.*^37^ for the application in fully automated animal surgery. In a recent work, Torun *et al.* proposed a drill breakthrough detection method based on parametric power spectral density estimation. By computing four frequency features and applying a neural network classifier they could reach a breakthrough detection accuracy of 92.37±1.09% in a 311.2${\hbox { ms}}$ time frame, using an artificial bone model^28^ and acoustic signals captured with an air-borne microphone.

Drill breakthrough detection and state classification based on acoustic signals has been shown to be a promising approach to supervise the surgical drilling process^28,31,32,34–36^. In prior work, studies have been conducted in an experimental setup using artificial bones or resected animal bone specimens and air-borne microphones attached to the drill or placed in close proximity to the area of operation. This is a limitation for the application in real surgery as the operating room is a noisy environment and the anatomy is not directly accessible because of surrounding soft tissue. To the best knowledge of the authors all previous studies implemented classical machine learning approaches, such as HMM, SVM or simple neural network classifiers. Recent advantages in deep learning methods for acoustic event classification have been shown to yield superior performances compared to classical approaches^38^. These approaches typically employ higher dimensional feature representations, such as spectrograms, which enable the deep network to learn the optimal features itself during the training process. Furthermore, typical window lengths in acoustic breakthrough detection of 300${\hbox { ms}}$, such as used in^28^, achieved promising results and have been applied for robotic drilling applications, where the robot is programmed with a slow feeding rate. However, they are not sufficient for free-hand drilling supervision, as a surgeon can react just as fast as the automated system^22^. To translate automated drill breakthrough detection into clinical use, adaptations to the hardware and data acquisition setup, as well as the development of a robust and fast classification method are crucial. The main goal of this approach is preventing surgical errors in form of over-drilling into the adjacent soft tissue, therefore increasing the safety of intervention and reducing patient risk.

## Cadaver experiments

In the following sections we will describe the experimental setup in detail, including recording hardware, conduction of the experiment and data preparation. Subsequently, the breakthrough detection method is introduced which is trained and validated on the dataset acquired in cadaver experiments.

### Low-cost contact microphone, impedance matching and amplification

Piezo-electric elements are made of crystalline material and produce small voltages when force or pressure is applied. This principle can, when amplified, be utilized to record vibrations as structure-borne sound from a surface by using piezo-electric elements as contact microphones. Structure-borne sounds have been shown to have great potential for analysis and information retrieval in medical applications^39^. Due to the physical nature of piezo-electric elements, the output impedance of the contact microphone lies typically in the range of several ${\hbox {M}\Omega }$. This results in an impedance mismatch with microphone or line inputs of recorders or mixers, which usually have an input impedance in the range of a few ${\hbox {k}\Omega }$. The mismatch results in high-pass filtering and poor transmission of signal energy in the low frequency region. Because we are interested in capturing also low-frequency components of the structure-borne vibration signal for breakthrough detection, an impedance matching stage is necessary. Furthermore, a high common-mode rejection ratio (CMRR) is desired to minimize electromagnetic interference. We use a 48${\hbox {V}}$ phantom-powered impedance matching circuit designed by Alex Rice (circuit design available under: https://www.zachpoff.com/resources/alex-rice-piezo-preamplifier/) and released under a Creative Commons Share-Alike 3.0 license. This circuit combines impedance-matching with a shielded and balanced connection, which suits the needs of our application. As contact sensor, we utilize a standard piezo disk with a diameter of 27mm. The contact microphone, impedance matching stage and analog/digital conversion stage are modular and connected through rugged XLR connector cables to allow different connection lengths for easy use.

**Figure 1:**
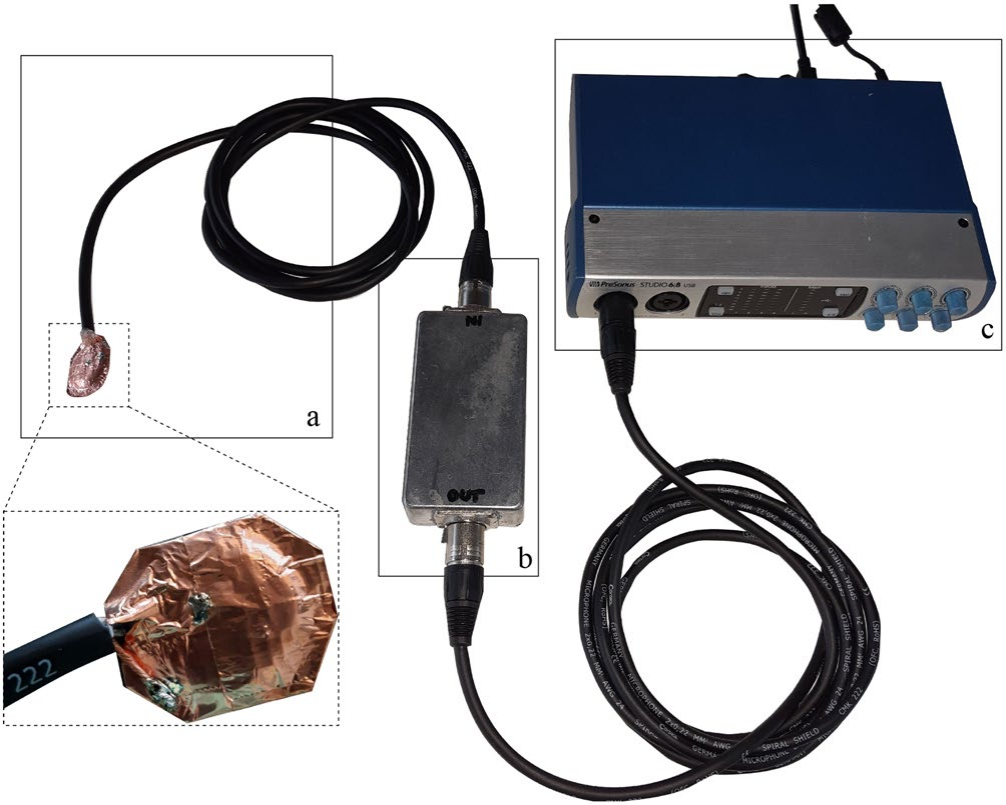
The recording chain consisting of (**a**) a shielded piezo contact microphone, (**b**) an impedance matching stage, and (**c**) an analog/digital conversion and amplification stage which allows to capture recordings from four sensors in parallel.

**Figure 2:**
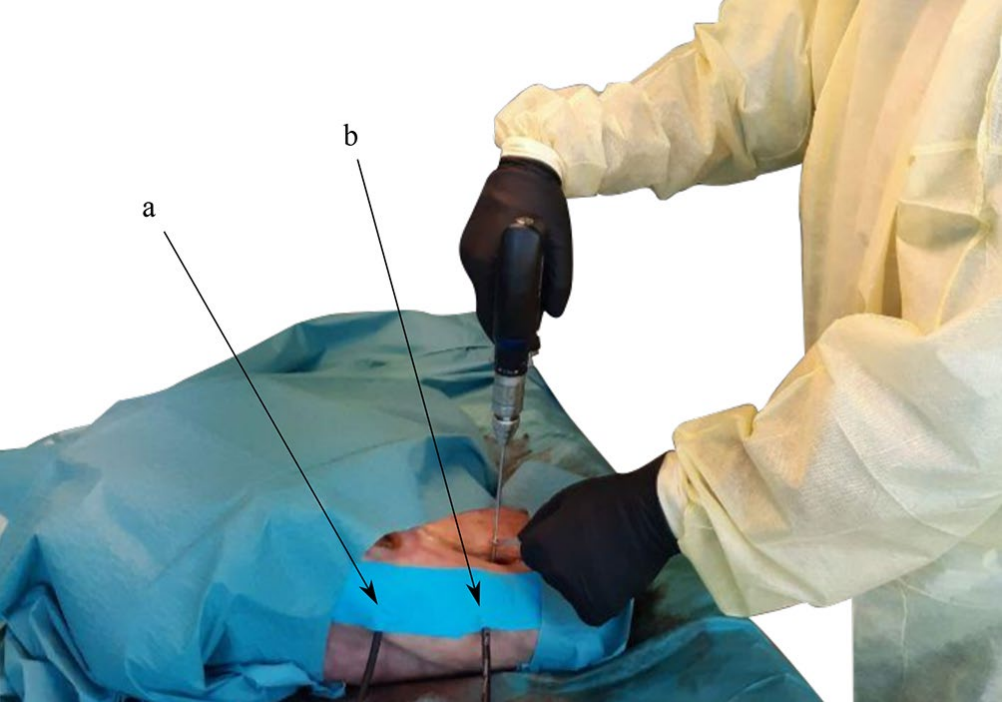
The experimental setup with a human cadaveric hip specimen. Two microphones are attached to the specimen’s skin surface with kinesiology tape to permit synchronously recording in parallel. The contact sensors are placed (**a**) at the *trochanter major* and (**b**) next to the incision, in *diaphysis* position.

To reduce the noise of the contact microphone and influence of electromagnetic fields, we electromagnetically shielded the entire circuit from piezo-element to the analog/digital converter and connected it to the system’s ground. Furthermore, before shielding, the piezo disk was covered in epoxy resin (WEICON GmbH & Co. KG, Münster, Germany) to make it rugged and avoid noise introduced by moving cables. Every cable connection (from piezo-element to impedance matching stage and from impedance matching stage to audio interface) is designed as balanced line to remove any electrical interference during signal transmission. This results in a highly sensitive and low-noise contact microphone which can be attached to the skin of patients to capture structure-borne signals. For amplification and analog/digital conversion we use the PreSonus Studio 68 (PreSonus Audio Electronics, Inc., Baton Rouge, LA, USA) audio interface and the Audio Stream Input/Output (ASIO) low-latency driver. The microphone amplifiers have a frequency response of 20 Hz–20 kHz with a tolerance of $\pm 0.15$ dB.

Another advantage of our setup is its modular design, which allows the contact microphone to be used as a disposable surgical instrument. All components of the contact microphone are low-cost ($<10$ USD) and therefore suited for single-use. The whole hardware setup can be built with an associated cost of about 300 USD. Figure 1 provides an overview of the recording chain which was used in the experiments described in the following section.

### Cadaver study design and data acquisition

To build an experimental setup which is as realistic and close to a clinical scenario as possible, we used three fresh frozen and unresected human cadaveric hip specimens to generate a dataset for training and validating the proposed network. An ethical approval for all ex-vivo experiments (Kantonale Ethikkommission Zurich, protocol number: 2020-01913), as well as informed consent from all subjects involved in this study and/or their legal guardians has been obtained. The experiments were conducted by a trained physician according to relevant guidelines and regulations. None of the cadavers used in our experiment had a record of previously assessed osteoporosis. The specimens were thawed, prepared, and incision in the lower extremities were made to access the area of operation at the proximal femur until the upper shaft of the anterior femur. For the surgical approach we used a direct transmuscular access for optimal presentation of the femur. The incisions were executed by intersecting the midline of the musculus quadriceps longitudinally with a scalpel and detaching it anteriorly from the surface of the femur. We then attached two contact microphones to the specimen’s skin surface using kinesiology tape. As illustrated in Fig. 2, one microphone was attached about 2${\hbox { cm}}$ next to the incision to minimize the distance that the acoustic waves propagate from source to microphone through the soft tissue, referred to as *diaphysis* position. The medical expert placed the second microphone on the skin where the *greater trochanter* is located. This placement was chosen because acoustic waves propagate well through bony tissue, a principle which has already been applied for bone quality assessment of long bones^40^, and the bone structure is easily identifiable for consistent placement in a clinical scenario. The effects of different sensor placement on drill breakthrough detection accuracy have been evaluated in this study as well and are presented in the section “Comparison of microphone positioning”.

We utilized a Colibri II battery powered drill (DePuy Synthes, Raynham, MA, USA) which is a standard power tool used in orthopedic surgery and a drill bit with a diameter of 3.2${\hbox { mm}}$ to drill holes into the femur. To create as realistic acoustic conditions as possible and to stabilize the drill on the periosteum, a tissue guard was used as seen in the Fig. 2. The drill bit was then placed in a right angle on the exposed periosteum of the anterior surface of the proximal femur and drilled in a continuous clockwise rotation. To be able to separate the recordings and assign them to the respective class, we recorded the breakthrough sequence from drilling through the second cortical layer of the femur until breaking through into the adjacent soft tissue. For each cadaver, we recorded data from both the left and right hip resulting in a total number of six individual bones. Overall, we captured audio recordings from 136 individual drill holes and respective breakthrough events in the experimental setup illustrated in Fig. 2. On average, about 22 holes were drilled in each femur, which corresponds to a realistic clinical scenario, as big Locking Compression Plates (LCP) plates with 20 and more holes exist for large bones.

After capturing, the recordings were manually cut, labelled and separated into two subsets $C:=\{cortical, \, breakthrough\}$, where $c_i$ denotes the respective class. We thoroughly identified each breakthrough sequence in the audio recordings by repeated acoustic and visual inspection in the respective spectrogram. In this context, the class $c_1$, *cortical*, contains samples of drilling cortical bone and the class $c_2$, *breakthrough*, contains samples of drill breakthrough events. All recordings processed within the digital audio workstation software REAPER. The samples were rendered without further application of software gain or processing. The recordings were captured with a sample rate of 44.1${\hbox { kHz}}$ and a bit depth of 24 bit. With a buffer size of 128 samples, the ASIO driver latency was measured as 6.8$\hbox { ms}$. In this configuration, up to four contact microphones can be recorded synchronously in parallel.

## Breakthrough detection method

### Pre-processing, feature extraction, data augmentation

Spectrogram features are the dominant representation in deep learning for audio signal processing^38^. They have been shown to yield superior classification performances and achieve promising results in combination with convolutional neural network-based architectures for speech^42^, audio event detection^43^, and medical applications^44^. Log-mel spectrograms, a widely-used spectrogram variant, are two-dimensional matrices with time windows as columns, mel-bins (frequency) as rows, and amplitude as scalar values contained in the matrix. Because of their grid-like regular structure, they are well suited to be processed using CNN classifiers. To compute log-mel spectrograms, the discrete signal was first segmented with a rectangular sliding window into short frames $x:[0:L-1]:=\{0,1,\ldots ,L-1\}\rightarrow {\mathbb R}$ of length *L* with 75% overlap. Short-Time Fourier Transformation (STFT) for each framed clip was computed using:

1 $$\begin{aligned}&X(m,k) = \sum _{n=0}^{N-1} x(n+mH)w(n) exp\left(\frac{-2\pi ikn}{N}\right), \quad where \end{aligned}$$

2 $$\begin{aligned}&w(n) = \frac{1}{2} \left[ 1- cos(2 \pi \frac{n}{N - 1})\right] , \quad n = 0,\ldots ,N-1 \end{aligned}$$

**Figure 3:**
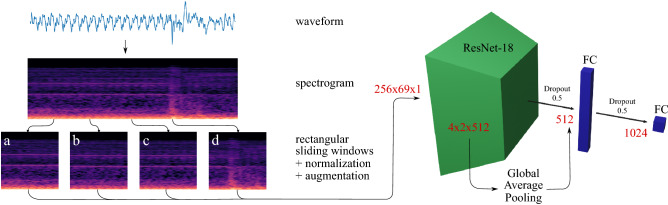
A breakthrough sequence with a total length of about 1${\hbox { s}}$, taken from the dataset acquired during the cadaver experiments. The raw waveform was split into frames by applying a rectangular sliding window and mel spectrogram features were computed. For better illustration, the spectrograms in this Figure are plotted using a colormap, however, the features used in the implementation of this work are two-dimensional only. Furthermore, the window length is chosen arbitrarily for better visualization and is not representative for the windows which have been evaluated in this work and are much shorter. Frames (**a**) to (**c**) correspond to the non-breakthrough class, in frame (**d**) the breakthrough event is present and visible in the spectrogram. The features were normalized and augmented which is described in detail in the section “Pre-processing, feature extraction, data augmentation”. A modified ResNet-18^41^ architecture, which is introduced in the section “Deep learning model and training”, was implemented to classify breakthrough events from spectrogram features. The output dimensions of each pipeline stage are given in red color.

Equation (2) denotes the *Hann* window function of length *N*, used for Eq. (1) to avoid *spectral leakage*^45^. The sliding window was shifted across the signal, using a step size specified by the parameter *H*, in samples. The resulting matrix *X* is a STFT spectrogram and contains the $k^{th}$ Fourier coefficient for the $m^{th}$ time frame.

To evaluate the performance of the proposed system in regard to the window length used for spectrogram generation, we implemented different hop lengths $H = \{64, 32, 16\}$ for the window lengths evaluated in this paper, $L = \{4410, 2205, 1102\}$, respectively, to keep the final spectrogram dimensions constant. The result was converted to a power spectrogram representation by squaring the amplitude and subsequently mapped to a logarithmic decibel scale by computing:

3 $$\begin{aligned} X_{pow}(m,k) = 10 \, log_{10}(X(m,k)_{2}) \end{aligned}$$

For transferring the matrix to the Mel scale, the result was filtered in the spectral domain with a triangular shaped Mel filter bank. The triangular filters are spaced evenly on the Mel scale which can be calculated from frequency with Eq. (4).

4 $$\begin{aligned} f_{mel} = 2595 \, log_{10}(1 + \frac{f}{700}) \end{aligned}$$

The log-mel spectrogram representation provides sparse, high resolution features for audio sources^46^. A total number of 256 Mel filter bands were applied to combine the Fast Fourier Transform (FFT) bins into Mel-frequency bins. All spectrograms were normalized by $X_{norm, mel} = {(X_{mel} - \mu )} / {\sigma }$, where ($\mu$) is mean and ($\sigma$) is the standard deviation computed over the entire training data.

Because the length of the breakthrough events is in the range of 100 to 250${\hbox { ms}}$ and much shorter compared to non-breakthrough sequences in our dataset, the number of spectrograms computed for the non-breakthrough class is more than an order of magnitude larger. This results in a highly unbalanced dataset. To balance the dataset, we use a data augmentation strategy and apply it to the underrepresented class by varying the gain (${-5}{\hbox {dB}}, {5}{\hbox {dB}}$), as well as applying time stretching (0.5, 0.7, 1.2, 1.5 times play rate) and pitch shifting ($-3$, $-1$, $+1$, $+3$ semitones) to the breakthrough event training samples.

A high-level overview of the pre-processing pipepline is illustrated in Fig. 3 in the left part of the Figure. All spectrograms were computed using the python library *librosa 0.7.2*^47^, are of size 256x69x1 and serve as input for the convolutional neural network architecture which is described in detail in the following paragraph.

### Deep learning model and training

The deep residual network (ResNet) architecture^41^ has been shown to perform exceptionally well on spectrogram-based audio classification tasks^48^. Because our aim is to develop a low-latency and reactive system we chose to implement a 18-layer ResNet variant which enables fast inference^49^. We found empirically to achieve the best results with a slightly modified architecture, stacking a global average pooling layer^50^, a dropout layer with a dropout rate of 0.5, a fully connected (FC) layer with 1024 neurons, another dropout and an output FC layer on top of ResNet-18’s final batch normalization layer. By introducing additional dropout layers for regularization, we reduce the model’s tendency towards overfitting. The final model has a total number of 11,715,393 parameters and its architecture is illustrated in Fig. 3.

To handle the problem of imbalanced data, we apply the *Focal Loss*^51^ as loss function for training. For imbalanced datasets, standard crossentropy is inefficient, as most samples fed to the network are classified with large confidence and therefore contribute no useful learning signal. The *Focal Loss* influences the network to focus on the underrepresented class which in our case corresponds to the breakthrough events that are crucial to detect with high accuracy for our particular application. The *Focal Loss* function is defined as:

5 $$\begin{aligned} FL(p_t) = -\alpha _t(1 - p_t)^\gamma \,log(p_t), \quad where \quad p_t = {\left\{ \begin{array}{ll} p \quad \quad \,\,\, \text {if}\, y = 1\\ 1-p \quad \text {otherwise} \end{array}\right. } \end{aligned}$$

The factors $\gamma$ and $\alpha _t$ are introduced as *focusing* and *balancing* parameter, respectively. In our implementation we use the by Lin *et al.* empirically determined optimal values $\alpha _t = 0.25$ and $\gamma = 2$^51^. The variable $p_t$ is defined for convenience, where *p* corresponds to the estimated probability for the class with label $y = 1$. We trained the model end-to-end on the spectrogram features explained in the section “Pre-processing, feature extraction, data augmentation” using the *Adam* optimizer and reduced the learning rate when stagnating loss was observed over three epochs by a factor of 10.

Model, training and inference were implemented using the open-source deep learning library *TensorFlow 2.2* and run on a NVIDIA GeForce RTX 2080 SUPER GPU. All results presented in the following sections have been evaluated using 5-fold cross-validation.

## Results

We split the evaluation section into three parts. First, we present the best performing variant of the proposed detection method and analyze the influence of design decisions on our detection pipeline. Afterwards, we compare the two microphone positions described in the section “Cadaver experiments” by analyzing the performance using synchronously acquired audio data. Subsequently, we evaluate different sliding window lengths *L* to assess the trade-off between detection latency and accuracy.

### Detection accuracy and performance

**Figure 4:**
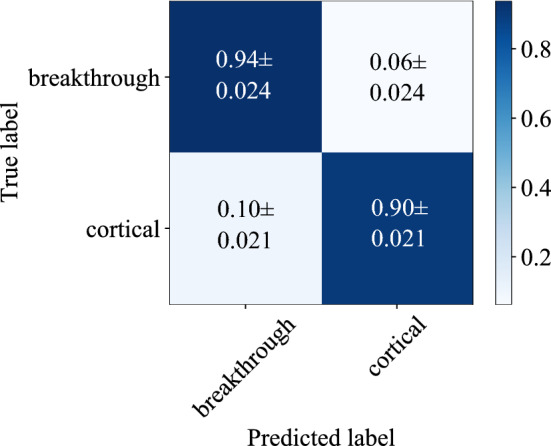
The normalized confusion matrix for a rectangular sliding window size of length $L = 4410$ samples which corresponds to a time frame of ${100}\hbox {{ms}}$, recorded in the *greater trochanter* position.

Figure 4 shows the confusion matrix for the best performing variant of our proposed algorithm, evaluated on the independent test set with a 100${\hbox { ms}}$ rectangular sliding window and for the data recorded in *greater trochanter* position. We measured a mean overall accuracy of $97.29 \%$ in the training and $91.90 \%$ in the test phase. The recall (sensitivity) of correctly detecting breakthrough events, which is the main performance measure for our application, is measured as $93.64 \pm 2.42$%.

**Figure 5:**
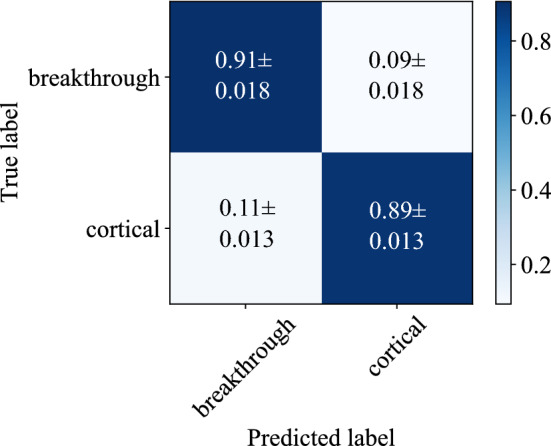
The normalized confusion matrix for a rectangular sliding window size of length $L = 4410$ samples which corresponds to a time frame of ${100}{\hbox {ms}}$, recorded in the *diaphysis* position.

Compared to the original ResNet-18 implementation^41^, we implemented several modifications which resulted in a performance gain. First, we modified the ResNet-18 architecture by including additional dropout and dense (FC) layers as described in the section “Deep learning model and training”. Through these modifications, we could boost the performance of the classification pipeline by about 3.5% for breakthrough recall. By implementing the *Focal Loss* as described in the section “Deep learning model and training” instead of the standard crossentropy loss, we could furthermore increase the model’s sensitivity for breakthrough event detection by 11.2%.

### Comparison of microphone positioning

We synchronously captured all recordings with two microphones in different positions as described in the section “Cadaver experiments”. One microphone was positioned directly above the *greater trochanter* to exploit the bone conductivity of acoustic waves. The second microphone was placed next to the incision (*diaphysis* position) to minimize the distance that the sound waves propagate through the soft tissue. The experimental setup and microphone positions are illustrated in Fig. 2. We treat the data acquired from the individual microphones as independent datasets to compare the microphone positions in regard to the resulting detection accuracy using a rectangular sliding window length of $L = 4410$ samples (100${\hbox { ms}}$).

In comparison to the results for the data recorded in *greater trochanter* position and illustrated in Fig. 4, it can be observed in Fig. 5 that positioning the microphone in *diaphysis* position yields inferior detection performance. The recall for detecting breakthrough events is lowered by roughly 3% to $90.61 \pm 1.77$%.

For evaluating the influence of the window length *L* in the following section, we focus on the dataset recorded by the microphone in the *greater trochanter* position.

### Comparison of window lengths

The length *L* of the rectangular sliding window determines the detection latency. With decreasing window length, the system is able to provide a detection result faster, as the audio chunk has to be acquired before it can be fed into the classification pipeline. However, the smaller the audio frame, the less information can be used by the network for feature extraction. We evaluated three window lengths, 100${\hbox { ms}}$, 50${\hbox { ms}}$ and 25${\hbox { ms}}$ to gain insights about the performance of the proposed pipeline in comparison to the latency. We did not evaluate larger window lengths, as the shortest samples of breakthrough events are only a few ${\hbox { ms}}$ longer than 100${\hbox { ms}}$.

To investigate the influence of shorter window lengths, we systematically reduced the window length and evaluated the detection performance which is illustrated in Table 1. By lowering the frame length, the sensitivity for breakthrough detection and for classification of non-breakthrough samples is reduced. In general, it can be observed that the model’s performance decreases with shorter window lengths.

**Table 1:** Comparison of window length.

Window length (ms)	Sensitivity breaktrough %	Sensitivity cortical %
25	$84.38 \pm 2.69$	$75.58 \pm 2.55$
50	$88.49 \pm 3.88$	$82.52 \pm 1.84$
100	$93.64 \pm 2.42$	$90.16 \pm 2.09$

**Figure 6:**
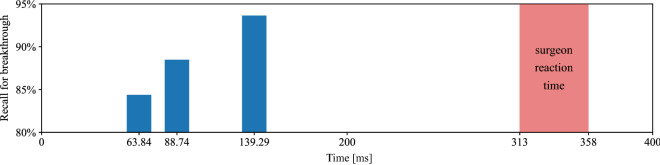
Pipeline execution speed and detection performance in comparison with surgeon reaction time, which has been measured to be in the range of 313 to 358${\hbox { ms}}$ and degrading with advancing age^22^. The execution times 63.84${\hbox { ms}}$, 88.74${\hbox { ms}}$ and 139.29${\hbox { ms}}$ correspond to window lengths of 25${\hbox { ms}}$, 50${\hbox { ms}}$ and 100${\hbox { ms}}$, respectively, as explained in the section “Comparison of window lengths”.

In Table 2, we show the measured execution time for each part of the proposed pipeline and the total execution time for one pass for a sample through the pipeline, given for different window lengths. All presented results have been averaged over 100 passes through the pipeline. Because we keep the spectrogram dimensions constant, spectrogram generation and ResNet-18 inference show very similar measured duration. We compare the above presented results to the average surgeon reaction time as measured in a previous study by Boom-Saad *et al.*^22^ in Fig. 6.

**Table 2:** Pipeline execution times.

Pipeline stage	Execution (ms)	Execution (ms)	Execution (ms)
ASIO driver latency	6.8	6.8	6.8
Window length	25	50	100
Spectrogram generation	7.01	6.92	6.98
ResNet-18 inference	25.03	25.02	25.51
Total execution time	63.84	88.74	139.29

## Discussion

Automated drill breakthrough detection is a promising approach for reducing the risk of surgical errors and iatrogenic injuries during the drilling process. To the best knowledge of the authors, our proposed method outperforms the results of all previous published work, for example Torun *et al.*, who used 300${\hbox { ms}}$ windows combined with handcrafted frequency features and achieved a detection sensitivity of $92.37 \pm 1.09$% for breakthrough detection in a simplified experimental setup based on a single artificial bone model^28^. Our best performing algorithm variant achieves a breakthrough detection sensitivity of $93.64 \pm 2.42$% using a 100${\hbox { ms}}$ window. We showed that our method performs well, even when reducing window lengths down to 25${\hbox { ms}}$. We are not only using much shorter window lengths, but also transferred breakthrough detection to pre-clinical experiments using human cadavers with soft tissues to mimic a realistic surgical intervention.

Low-latency detection is crucial to stop the drill as fast as possible when a breakthrough event is observed. The total latency of the best performing variant of our algorithm amounts to 139.29${\hbox { ms}}$. However, by reducing the window length we still achieve sensitivities of $88.49 \pm 3.88$% in 88.74${\hbox { ms}}$ and $84.38 \pm 2.69$% in 63.84${\hbox { ms}}$ for breakthrough detection. The larger the observed window, the more temporal context is provided to the model as basis for feature extraction and classification. As the performance of our solution still reaches fairly high classification accuracies with short window length, the trade-off between accuracy and latency has to be chosen for the particular application. The detection speed of our pipeline clearly outperforms the human reaction time and has therefore great potential to increase the safety during drilling in surgery .

Using easy-to-integrate contact microphones, we acquired structure-borne audio signals during drilling execution directly from the skin surface with very little noise disturbances. Acquiring a dataset in a realistic scenario such as cadaveric experiments has the advantage of capturing realistic characteristics of structure-borne acoustic signal dampening through soft tissue which is not possible to simulate with artificial bone models or prepared bones. Concerning the positioning of the contact microphones, our results show that the sounds captured in *greater trochanter* position yield better classification performance, compared to placing the microphone close to the incision in the *diaphysis* position. By exploiting the bone conductivity of acoustic waves and at the same time providing a reproducible positioning of the microphone, the *greater trochanter* position is optimal for the task of drill breakthrough detecting with contact sensors in hip surgery.

We believe that the deep-learning based analysis of structure-borne acoustic signals is a promising approach to supervise the surgical drilling process and that the proposed solution paves the path for deployment and testing the approach in real surgery. However, to translate the proposed system into clinical use in the operating room, the following limitations of the presented study have to be overcome. A clinical study is necessary to evaluate the reliability and robustness of the solution in-vivo. Furthermore, the performance of the algorithm could potentially be further improved by increasing the size of the dataset to expand the model’s capability for generalization, including multiple surgeons and different anatomies. The proposed hardware setup, illustrated in detail in Fig. 1, is modular, low-cost and the contact microphones can be replaced easily. However, the the sterilizability of the electrically shielded contact sensor (part *a* in Fig. 1) has to be investigated and validated. We did not measure the frequency transmission characteristics of the deployed circuitry explicitly, however the presented configuration enables high quality and low-noise audio recordings and increased bandwidth of the piezo element through impedance matching. Even though we thoroughly labelled each breakthrough sequence in the audio recordings by repeated acoustic (with professional studio-grade headphones) and visual inspection in the respective spectrogram (with high resolution in time), small uncertainties in the ground truth labelling process cannot be ruled out.

Currently, our system is running on a development computer, using high-end and high power hardware. To transfer the developed solution to an embedded solution, strategies such as model quantization can be employed to decrease the model size and resource requirements^52^. In addition, it is crucial to stop the drill as soon as a breakthrough event is detected to reliably increase the safety of surgical procedures by automated drill breakthrough detection. To this end, a stopping mechanism or circuitry has to be integrated into the drill which should be able to stop the drill with as minimal additional latency as possible.

## Conclusion

In this paper, we present a deep-learning based approach for automated drill breakthrough detection in orthopedic interventions using acoustic emission signals. We developed a hardware setup employing piezo-based contact microphones to capture vibration signals non-invasively from the skin surface. The proposed experimental setup was utilized to capture a dataset of drill vibration signals from six human cadaveric hips.

Our classification pipeline reaches a sensitivity of $93.64 \pm 2.42$% on the task of drill breakthrough detection, in a total execution time of 139.29${\hbox { ms}}$. Faster versions of our solution yield a sensitivity of $88.49 \pm 3.88$% in 88.74${\hbox { ms}}$ and $84.38 \pm 2.69$% in 63.84${\hbox { ms}}$ execution time. We show, that the proposed system is able to detect breakthrough events with high accuracy while being multiple times faster than the reaction time of trained surgeons. In addition, we evaluated different positioning of the contact sensors and observed that best results can be obtained by exploiting the conductivity of acoustic waves through bone tissue and placing the microphone as close as possible to subcutaneous bony structures.

The proposed solution has great potential to be used as a system for error prevention in surgery by preventing a damage to soft tissue and vital adjacent structures during bone drilling. Because drilling is an essential part in the vast majority of orthopedic interventions, the proposed system could have a great impact on patient safety and surgery outcome. Our exemplary application shows that acoustic sensing offers a very accurate, easy-to-integrate and low-cost approach to prevent errors in surgery which can be easily transferred to other surgical applications.
