# Author Correction: Real‑time acoustic sensing and artificial intelligence for error prevention in orthopedic surgery

**DOI:** 10.1038/s41598-021-89241-0

**Published:** 2021-04-29

**Authors:** Matthias Seibold, Steven Maurer, Armando Hoch, Patrick Zingg, Mazda Farshad, Nassir Navab, Philipp Fürnstahl

**Affiliations:** 1 Computer Aided Medical Procedures (CAMP), Technical University of Munich, 85748 Munich, Germany; 2 Research in Orthopedic Computer Science (ROCS), University Hospital Balgrist, University of Zurich, Balgrist Campus, 8008 Zurich, Switzerland; 3 Balgrist University Hospital, 8008 Zurich, Switzerland

Correction to: *Scientific Reports* https://doi.org/10.1038/s41598-021-83506-4, published online 17 February 2021

The original version of this Article contained errors in affiliations for authors Nassir Navab and Philipp Fürnstahl.

Nassir Navab was incorrectly affiliated with ‘Research in Orthopedic Computer Science (ROCS), University Hospital Balgrist, University of Zurich, Balgrist Campus, 8008 Zurich, Switzerland’. The correct affiliation is listed below:

Computer Aided Medical Procedures (CAMP), Technical University of Munich, 85748 Munich, Germany.

Philipp Fürnstahl was incorrectly affiliated with ‘Computer Aided Medical Procedures (CAMP), Technical University of Munich, 85748 Munich, Germany’. The correct affiliations are listed below:

Research in Orthopedic Computer Science (ROCS), University Hospital Balgrist, University of Zurich, Balgrist Campus, 8008 Zurich, Switzerland.

Balgrist University Hospital, 8008, Zurich, Switzerland.

This has now been corrected in the HTML and PDF versions of this Article.

